# *N*′-[(*E*)-Furan-2-ylmethyl­idene]-4-hydroxy­benzohydrazide

**DOI:** 10.1107/S1600536814001822

**Published:** 2014-02-05

**Authors:** Riya Datta, V. Ramya, M. Sithambaresan, M. R. Prathapachandra Kurup

**Affiliations:** a Department of Chemistry, Christ University, Hosur Road, Bangalore 560 029, India; b Department of Chemistry, Faculty of Science, Eastern University, Sri Lanka, Chenkalady, Sri Lanka; c Department of Applied Chemistry, Cochin University of Science and Technology, Kochi 682 022, India

## Abstract

The title compound, C_12_H_10_N_2_O_3_, exists in the *E* conformation. The five-membered ring and the phenyl rings form dihedral angles of 36.73 (10) and 12.22 (10)°, respectively, with the central C(=O)N_2_C unit. The crystal packing is dominated by strong N—H⋯O and O—H⋯N hydrogen bonds. Together with weaker C—H⋯O inter­actions, these establish a three-dimensional supra­molecular network.

## Related literature   

For biological applications of benzohydrazones and derivatives, see: Sreeja *et al.* (2004 ▶); Rakha *et al.* (1996 ▶). For the synthesis of related compounds, see: Emmanuel *et al.* (2011 ▶). For a related structure, see: Datta *et al.* (2013 ▶).
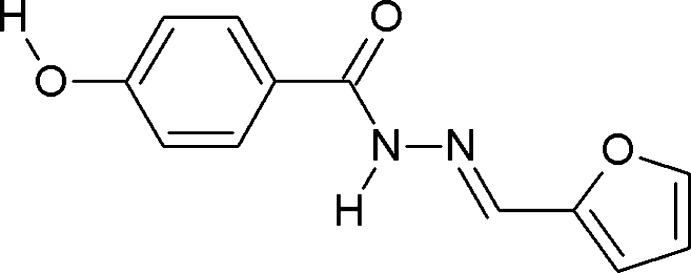


## Experimental   

### 

#### Crystal data  

C_12_H_10_N_2_O_3_
*M* *_r_* = 230.22
Orthorhombic, 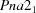

*a* = 9.5934 (3) Å
*b* = 11.1939 (4) Å
*c* = 10.3332 (3) Å
*V* = 1109.66 (6) Å^3^
*Z* = 4
Mo *K*α radiation
μ = 0.10 mm^−1^
*T* = 298 K
0.25 × 0.20 × 0.16 mm

#### Data collection  

Bruker APEXII CCD area-detector diffractometer
Absorption correction: multi-scan (*SADABS*; Bruker, 2004 ▶) *T*_min_ = 0.975, *T*_max_ = 0.984
3425 measured reflections
1014 independent reflections
992 reflections with *I* > 2σ(*I*)
*R*_int_ = 0.015

#### Refinement  

*R*[*F* ^2^ > 2σ(*F* ^2^)] = 0.024
*wR*(*F* ^2^) = 0.064
*S* = 1.05
1014 reflections
163 parameters
3 restraints
H atoms treated by a mixture of independent and constrained refinement
Δρ_max_ = 0.13 e Å^−3^
Δρ_min_ = −0.10 e Å^−3^

### 

Data collection: *APEX2* (Bruker, 2004 ▶); cell refinement: *APEX2* and *SAINT* (Bruker, 2004 ▶); data reduction: *SAINT* and *XPREP* (Bruker, 2004 ▶); program(s) used to solve structure: *SHELXS97* (Sheldrick, 2008 ▶); program(s) used to refine structure: *SHELXL97* (Sheldrick, 2008 ▶); molecular graphics: *ORTEP-3 for Windows* (Farrugia, 2012 ▶) and *DIAMOND* (Brandenburg, 2010 ▶); software used to prepare material for publication: *SHELXL97* and *publCIF* (Westrip, 2010 ▶).

## Supplementary Material

Crystal structure: contains datablock(s) Global, I. DOI: 10.1107/S1600536814001822/fj2657sup1.cif

Structure factors: contains datablock(s) I. DOI: 10.1107/S1600536814001822/fj2657Isup2.hkl

Supporting information file. DOI: 10.1107/S1600536814001822/fj2657Isup3.cml

CCDC reference:

Additional supporting information:  crystallographic information; 3D view; checkCIF report

## Figures and Tables

**Table 1:** Hydrogen-bond geometry (Å, °)

*D*—H⋯*A*	*D*—H	H⋯*A*	*D*⋯*A*	*D*—H⋯*A*
N2—H2′⋯O2^i^	0.88 (1)	2.09 (1)	2.9187 (19)	157 (2)
O3—H3′⋯N1^ii^	0.85 (1)	2.13 (1)	2.971 (2)	169 (3)
C5—H5⋯O2^i^	0.93	2.35	3.160 (2)	145
C11—H11⋯O3^iii^	0.93	2.42	3.202 (2)	142

Symmetry codes: (i) 
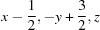
; (ii) 
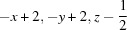
; (iii) 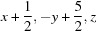.

**Fig. 1.**
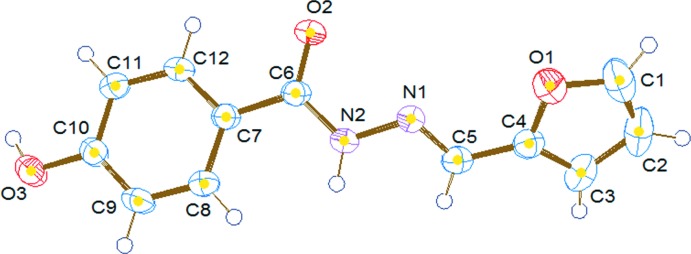
ORTEP view of the title compound drawn with 50% probability displacement ellipsoids for the non-H atoms.

**Fig. 2.**
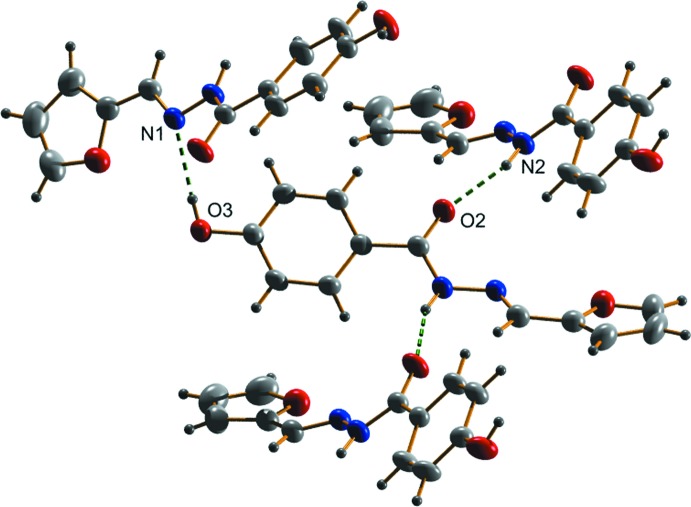
Classical hydrogen-bonding interactions in the crystal structure of C12H10N2O3.

**Fig. 3.**
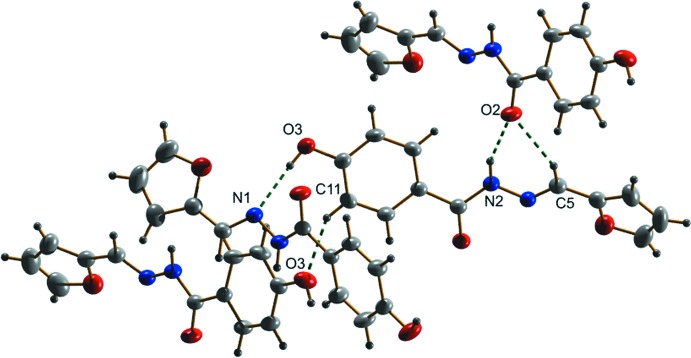
Hydrogen-bonding interactions in the crystal structure of C12H10N2O3.

**Fig. 4.**
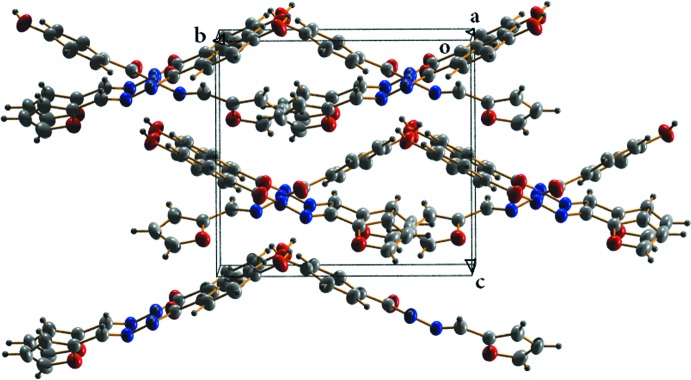
Packing diagram of the compound along the a axis.

## supplementary crystallographic information

### 1. Comment

Hydrazones and their derivatives show excellent biological activities (Sreeja *et al.*, 2004). The great potential applications of aryl- hydrazones as antineoplastic, antiviral and antiinflammatory agents, hammered on the investigations of their derivatives (Rakha *et al.*, 1996). As a continuous work on hydrazone compounds, a new hydrazone derivative, *N*'-[(*E*)-4,5-dihydrofuran-2-ylmethylidene]-4-hydroxybenzohydrazide, was prepared and structurally characterized. The *ORTEP* view of the title compound is shown in Fig. 1.

The compound crystallizes in orthorhombic space group *Pna*2_1_. This molecule adopts an *E* configuration with respect to the C5=N1 bond and it exists in the amido form with a C6=O2 bond length of 1.232 (2) Å which is very close to the reported C=O bond length of similar structure (Datta *et al.*, 2013). The O2 and N1 atoms are in *Z* configuration with respect to C6–N2 having a torsion angle of 3.7 (3)°. The central C(=O)N_2_C unit has dihedral angles of 36.73 (10) and 12.22 (10)°, respectively with the five-membered ring and the phenyl ring.

There are two classical intermolecular N2–H2'···O2 and O3–H3'···N1 hydrogen bond interactions (Fig. 2) between the neighbouring molecule with D···A distances of 2.9187 (19) and 2.971 (2) Å respectively (Table 1). Two weak C–H···O hydrogen bond interactions (Fig. 3) between the H atoms attached at the C5 & C11 and O2 & O3 atoms of neighbouring molecules with D···A distances of 3.160 (2) and 3.202 (2) Å respectively, also promote the classical hydrogen bond interactions forming a supramolecular three-dimensional-hydrogen bonding network in the lattice. Notwithstanding that there are very weak short ring interactions found in the crystal system, they are not significant to support the network since centroid-centroid distances are above 4 Å. Fig. 4 shows a packing diagram of the title compound viewed along *a* axis.

### 2. Experimental

The title compound was prepared by adapting a reported procedure (Emmanuel *et al.*, 2011). A solution of furan-2-carbaldehyde (0.096 g, 1 mmol) in methanol/DMF 2:1 (10 ml) was mixed with a methanol/DMF solution (10 ml) of 4-hydroxybenzhydrazide (0.152 g, 1 mmol). The mixture was refluxed for 6 h and then cooled to room temperature. Light orange colored crystals were formed which were recrystallized in methanol/DMF (2:1 *v*/*v*). Block shaped crystals, suitable for SXRD studies, were obtained after slow evaporation of the solution in air for a few days.

### 3. Refinement

The atoms H2' and H3' were located from a difference Fourier map and N2—H2' and O3—H3' distances are restrained to 0.88±0.01 and 0.84±0.01 Å respectively. All the other H atoms on C were placed in calculated positions, guided by difference maps, with C–H bond distances 0.93 Å. H atoms were assigned as *U*_iso_(H)=1.2Ueq(carrier).

### Crystal data

**Table:**

C_12_H_10_N_2_O_3_	*F*(000) = 480
*M**_r_* = 230.22	*D*_x_ = 1.378 Mg m^−^^3^
Orthorhombic, *P**n**a*2_1_	Mo *K*α radiation, λ = 0.71073 Å
Hall symbol: P 2c -2n	θ = 2.7–28.4°
*a* = 9.5934 (3) Å	µ = 0.10 mm^−^^1^
*b* = 11.1939 (4) Å	*T* = 298 K
*c* = 10.3332 (3) Å	Block, light orange
*V* = 1109.66 (6) Å^3^	0.25 × 0.20 × 0.16 mm
*Z* = 4	

### Data collection

**Table:**

Bruker APEXII CCD area-detector diffractometer	1014 independent reflections
Radiation source: fine-focus sealed tube	992 reflections with *I* > 2σ(*I*)
Graphite monochromator	*R*_int_ = 0.015
Detector resolution: 8.33 pixels mm^-1^	θ_max_ = 25.0°, θ_min_ = 2.7°
phi and ω scans	*h* = −11→11
Absorption correction: multi-scan (*SADABS*; Bruker, 2004)	*k* = −13→9
*T*_min_ = 0.975, *T*_max_ = 0.984	*l* = −10→12
3425 measured reflections	

### Refinement

**Table:**

Refinement on *F*^2^	Secondary atom site location: difference Fourier map
Least-squares matrix: full	Hydrogen site location: inferred from neighbouring sites
*R*[*F*^2^ > 2σ(*F*^2^)] = 0.024	H atoms treated by a mixture of independent and constrained refinement
*wR*(*F*^2^) = 0.064	*w* = 1/[σ^2^(*F*_o_^2^) + (0.0363*P*)^2^ + 0.1774*P*] where *P* = (*F*_o_^2^ + 2*F*_c_^2^)/3
*S* = 1.05	(Δ/σ)_max_ < 0.001
1014 reflections	Δρ_max_ = 0.13 e Å^−^^3^
163 parameters	Δρ_min_ = −0.10 e Å^−^^3^
3 restraints	Extinction correction: *SHELXL97* (Sheldrick, 2008), Fc^*^=kFc[1+0.001xFc^2^λ^3^/sin(2θ)]^-1/4^
Primary atom site location: structure-invariant direct methods	Extinction coefficient: 0.046 (5)

### Special details

**Table:**

Geometry. All e.s.d.'s (except the e.s.d. in the dihedral angle between two l.s. planes) are estimated using the full covariance matrix. The cell e.s.d.'s are taken into account individually in the estimation of e.s.d.'s in distances, angles and torsion angles; correlations between e.s.d.'s in cell parameters are only used when they are defined by crystal symmetry. An approximate (isotropic) treatment of cell e.s.d.'s is used for estimating e.s.d.'s involving l.s. planes.
Refinement. Refinement of *F*^2^ against ALL reflections. The weighted *R*-factor *wR* and goodness of fit *S* are based on *F*^2^, conventional *R*-factors *R* are based on *F*, with *F* set to zero for negative *F*^2^. The threshold expression of *F*^2^ > σ(*F*^2^) is used only for calculating *R*-factors(gt) *etc*. and is not relevant to the choice of reflections for refinement. *R*-factors based on *F*^2^ are statistically about twice as large as those based on *F*, and *R*- factors based on ALL data will be even larger.

### Fractional atomic coordinates and isotropic or equivalent isotropic displacement parameters (Å^2^)

**Table:**

	*x*	*y*	*z*	*U*_iso_*/*U*_eq_	
O1	1.10537 (16)	0.44042 (12)	0.85962 (16)	0.0487 (4)	
O2	1.15292 (13)	0.82491 (12)	0.64988 (16)	0.0454 (4)	
O3	0.79142 (15)	1.25293 (13)	0.43449 (17)	0.0509 (4)	
N1	1.00354 (16)	0.64857 (13)	0.74734 (17)	0.0346 (4)	
N2	0.94150 (15)	0.74797 (14)	0.69195 (18)	0.0357 (4)	
C1	1.1354 (3)	0.3230 (2)	0.8813 (3)	0.0609 (7)	
H1	1.2119	0.2956	0.9279	0.073*	
C2	1.0401 (3)	0.2529 (2)	0.8265 (3)	0.0655 (7)	
H2	1.0390	0.1698	0.8271	0.079*	
C3	0.9410 (3)	0.32850 (19)	0.7673 (3)	0.0528 (6)	
H3	0.8614	0.3054	0.7224	0.063*	
C4	0.9853 (2)	0.44092 (17)	0.7889 (2)	0.0394 (5)	
C5	0.9330 (2)	0.55207 (17)	0.7401 (2)	0.0389 (5)	
H5	0.8452	0.5539	0.7019	0.047*	
C6	1.02519 (18)	0.83589 (16)	0.64733 (19)	0.0332 (4)	
C7	0.95845 (19)	0.94475 (16)	0.5952 (2)	0.0327 (4)	
C8	0.81588 (19)	0.96915 (18)	0.6015 (2)	0.0422 (5)	
H8	0.7566	0.9156	0.6428	0.051*	
C9	0.7622 (2)	1.07164 (18)	0.5474 (2)	0.0464 (5)	
H9	0.6670	1.0866	0.5525	0.056*	
C10	0.8484 (2)	1.15259 (16)	0.48562 (19)	0.0363 (5)	
C11	0.9907 (2)	1.13042 (17)	0.4805 (2)	0.0400 (5)	
H11	1.0502	1.1850	0.4410	0.048*	
C12	1.04335 (19)	1.02759 (17)	0.5340 (2)	0.0383 (5)	
H12	1.1387	1.0131	0.5291	0.046*	
H2'	0.8518 (11)	0.7474 (18)	0.676 (2)	0.041 (6)*	
H3'	0.853 (2)	1.288 (2)	0.389 (2)	0.062 (8)*	

### Atomic displacement parameters (Å^2^)

**Table:**

	*U*^11^	*U*^22^	*U*^33^	*U*^12^	*U*^13^	*U*^23^
O1	0.0473 (8)	0.0475 (9)	0.0515 (9)	0.0037 (7)	−0.0047 (7)	0.0042 (8)
O2	0.0233 (6)	0.0449 (8)	0.0679 (10)	0.0008 (5)	−0.0012 (7)	0.0128 (8)
O3	0.0378 (8)	0.0491 (9)	0.0657 (11)	0.0131 (7)	0.0115 (9)	0.0196 (8)
N1	0.0280 (7)	0.0328 (8)	0.0428 (9)	0.0011 (6)	0.0003 (7)	0.0026 (7)
N2	0.0235 (7)	0.0349 (8)	0.0488 (10)	0.0005 (6)	−0.0024 (8)	0.0037 (7)
C1	0.0719 (16)	0.0551 (15)	0.0556 (14)	0.0205 (13)	0.0011 (14)	0.0138 (12)
C2	0.098 (2)	0.0364 (12)	0.0621 (15)	0.0059 (13)	0.0102 (16)	0.0116 (12)
C3	0.0609 (14)	0.0396 (11)	0.0578 (14)	−0.0100 (10)	0.0026 (12)	0.0031 (11)
C4	0.0360 (9)	0.0397 (10)	0.0426 (11)	−0.0017 (8)	0.0032 (9)	0.0006 (9)
C5	0.0302 (9)	0.0388 (10)	0.0478 (12)	−0.0020 (7)	−0.0008 (10)	−0.0004 (9)
C6	0.0261 (8)	0.0356 (9)	0.0380 (10)	−0.0006 (7)	−0.0014 (8)	−0.0012 (8)
C7	0.0271 (9)	0.0337 (9)	0.0372 (10)	−0.0006 (7)	−0.0001 (9)	−0.0020 (8)
C8	0.0283 (9)	0.0430 (10)	0.0554 (12)	0.0004 (8)	0.0087 (10)	0.0099 (11)
C9	0.0260 (9)	0.0519 (12)	0.0614 (13)	0.0090 (8)	0.0089 (11)	0.0101 (12)
C10	0.0328 (10)	0.0362 (10)	0.0399 (12)	0.0054 (8)	0.0017 (9)	0.0015 (9)
C11	0.0295 (10)	0.0416 (10)	0.0488 (13)	−0.0036 (8)	0.0037 (9)	0.0084 (10)
C12	0.0227 (9)	0.0419 (10)	0.0504 (13)	0.0010 (7)	0.0001 (9)	0.0044 (9)

### Geometric parameters (Å, º)

**Table:**

O1—C4	1.364 (3)	C3—H3	0.9300
O1—C1	1.364 (3)	C4—C5	1.433 (3)
O2—C6	1.232 (2)	C5—H5	0.9300
O3—C10	1.356 (2)	C6—C7	1.478 (2)
O3—H3'	0.847 (10)	C7—C12	1.387 (2)
N1—C5	1.277 (2)	C7—C8	1.396 (3)
N1—N2	1.386 (2)	C8—C9	1.376 (3)
N2—C6	1.351 (2)	C8—H8	0.9300
N2—H2'	0.876 (10)	C9—C10	1.383 (3)
C1—C2	1.331 (4)	C9—H9	0.9300
C1—H1	0.9300	C10—C11	1.389 (3)
C2—C3	1.412 (4)	C11—C12	1.373 (3)
C2—H2	0.9300	C11—H11	0.9300
C3—C4	1.347 (3)	C12—H12	0.9300
			
C4—O1—C1	105.67 (19)	O2—C6—N2	120.73 (17)
C10—O3—H3'	108.7 (18)	O2—C6—C7	121.39 (16)
C5—N1—N2	115.31 (16)	N2—C6—C7	117.87 (15)
C6—N2—N1	118.08 (14)	C12—C7—C8	117.77 (17)
C6—N2—H2'	121.6 (14)	C12—C7—C6	117.59 (15)
N1—N2—H2'	119.6 (14)	C8—C7—C6	124.63 (17)
C2—C1—O1	110.7 (2)	C9—C8—C7	120.71 (19)
C2—C1—H1	124.7	C9—C8—H8	119.6
O1—C1—H1	124.7	C7—C8—H8	119.6
C1—C2—C3	107.1 (2)	C8—C9—C10	120.64 (17)
C1—C2—H2	126.5	C8—C9—H9	119.7
C3—C2—H2	126.5	C10—C9—H9	119.7
C4—C3—C2	106.0 (2)	O3—C10—C9	118.77 (17)
C4—C3—H3	127.0	O3—C10—C11	121.97 (18)
C2—C3—H3	127.0	C9—C10—C11	119.24 (18)
C3—C4—O1	110.59 (19)	C12—C11—C10	119.74 (18)
C3—C4—C5	129.9 (2)	C12—C11—H11	120.1
O1—C4—C5	119.19 (18)	C10—C11—H11	120.1
N1—C5—C4	121.89 (19)	C11—C12—C7	121.88 (16)
N1—C5—H5	119.1	C11—C12—H12	119.1
C4—C5—H5	119.1	C7—C12—H12	119.1
			
C5—N1—N2—C6	−152.8 (2)	N2—C6—C7—C12	−171.64 (19)
C4—O1—C1—C2	0.4 (3)	O2—C6—C7—C8	−172.9 (2)
O1—C1—C2—C3	−0.9 (3)	N2—C6—C7—C8	7.4 (3)
C1—C2—C3—C4	1.0 (3)	C12—C7—C8—C9	0.7 (3)
C2—C3—C4—O1	−0.8 (3)	C6—C7—C8—C9	−178.4 (2)
C2—C3—C4—C5	173.0 (2)	C7—C8—C9—C10	0.0 (4)
C1—O1—C4—C3	0.2 (3)	C8—C9—C10—O3	−179.5 (2)
C1—O1—C4—C5	−174.3 (2)	C8—C9—C10—C11	−1.1 (3)
N2—N1—C5—C4	177.85 (18)	O3—C10—C11—C12	179.84 (19)
C3—C4—C5—N1	−164.8 (3)	C9—C10—C11—C12	1.5 (3)
O1—C4—C5—N1	8.5 (3)	C10—C11—C12—C7	−0.8 (3)
N1—N2—C6—O2	3.7 (3)	C8—C7—C12—C11	−0.3 (3)
N1—N2—C6—C7	−176.57 (16)	C6—C7—C12—C11	178.9 (2)
O2—C6—C7—C12	8.1 (3)		

### Hydrogen-bond geometry (Å, º)

**Table:**

*D*—H···*A*	*D*—H	H···*A*	*D*···*A*	*D*—H···*A*
N2—H2′···O2^i^	0.88 (1)	2.09 (1)	2.9187 (19)	157 (2)
O3—H3′···N1^ii^	0.85 (1)	2.13 (1)	2.971 (2)	169 (3)
C5—H5···O2^i^	0.93	2.35	3.160 (2)	145
C11—H11···O3^iii^	0.93	2.42	3.202 (2)	142

Symmetry codes: (i) *x*−1/2, −*y*+3/2, *z*; (ii) −*x*+2, −*y*+2, *z*−1/2; (iii) *x*+1/2, −*y*+5/2, *z*.
